# Innate and Adaptive Immune Assessment at Admission to Predict Clinical Outcome in COVID-19 Patients

**DOI:** 10.3390/biomedicines9080917

**Published:** 2021-07-29

**Authors:** David San Segundo, Francisco Arnáiz de las Revillas, Patricia Lamadrid-Perojo, Alejandra Comins-Boo, Claudia González-Rico, Marta Alonso-Peña, Juan Irure-Ventura, José Manuel Olmos, María Carmen Fariñas, Marcos López-Hoyos

**Affiliations:** 1 Immunology Service, University Hospital Marqués de Valdecilla, 39008 Santander, Spain; david.sansegundo@scsalud.es (D.S.S.); alejandra.comins@scsalud.es (A.C.-B.); juan.irure@scsalud.es (J.I.-V.); 2 Transplantation and Autoimmunity Laboratory, Research Institute “Marqués de Valdecilla” (IDIVAL), 39011 Santander, Spain; plamadrid@idival.org (P.L.-P.); malonso@idival.org (M.A.-P.); 3 Infectious Diseases Service, University Hospital Marqués de Valdecilla, 39008 Santander, Spain; francisco.arnaizlasrevillas@scsalud.es (F.A.d.l.R.); claudia.gonzalez@scsalud.es (C.G.-R.); mcarmen.farinas@scsalud.es (M.C.F.); 4 Internal Medicine Service, University Hospital Marqués de Valdecilla, 39008 Santander, Spain; josemanuel.olmos@scsalud.es; 5 Faculty of Medicine, University of Cantabria, 39011 Santander, Spain

**Keywords:** SARS-CoV-2, flow cytometry, innate immunity, adaptive immunity, immunological profile, predictive model

## Abstract

During the COVID-19 pandemic, many studies have been carried out to evaluate different immune system components to search for prognostic biomarkers of the disease. A broad multiparametric antibody panel of cellular and humoral components of the innate and the adaptative immune response in patients with active SARS-CoV-2 infection has been evaluated in this study. A total of 155 patients were studied at admission into our center and were categorized according to the requirement of oxygen therapy as mild or severe (the latter being those with the requirement). The patients with severe disease were older and had high ferritin, D-dimer, C-reactive protein, troponin, interleukin-6 (IL-6) levels, and neutrophilia with lymphopenia at admission. Moreover, the patients with mild symptoms had significantly increased circulating non-classical monocytes, innate lymphoid cells, and regulatory NK cells. In contrast, severe patients had a low frequency of Th1 and regulatory T cells with increased activated and exhausted CD8 phenotype (CD8^+^CD38^+^HLADR^+^ and CD8^+^CD27^−^CD28^−^, respectively). The predictive model included age, ferritin, D-dimer, lymph counts, C4, CD8^+^CD27^−^CD28^−^, and non-classical monocytes in the logistic regression analysis. The model predicted severity with an area under the curve of 78%. Both innate and adaptive immune parameters could be considered potential predictive biomarkers of the prognosis of COVID-19 disease.

## 1. Introduction

COVID-19 is an infectious disease induced by the novel coronavirus SARS-CoV-2 first detected in December 2019, causing acute respiratory distress syndrome (ARDS). Due to its high rate of transmission, it has reached pandemic status. The clinical picture of the infection ranges from asymptomatic or mildly symptomatic to lethal, mainly affecting the elderly population and those with associated comorbidities [1,2].

Early after COVID-19 breakout, different parameters were identified as prognostic markers of death, such as serum D-dimer, IL-6, troponin, ferritin, lactate dehydrogenase (LDH), and lymph count [3]. Subsequently, several groups worldwide confirmed these parameters and proposed new factors at admission to identify those patients with poor outcomes [4,5,6,7,8].

The variability of the antiviral immune response in healthy subjects might underlie the diverse array of clinical manifestations. Moreover, therapeutic approaches, primarily based on previous SARS, MERS, and inflammatory disorders associated with the cytokine storm, demonstrate different efficacies.

Considering that SARS-CoV-2 is a foreign invader in our organism, the immune response seems vital in clearing the infection. Furthermore, a dysregulated immune response appears to play a crucial role in the second phase of the disease, which manifests itself in intensive care units and might result in death [9].

Circulating immune cells and soluble immune components can be detected in peripheral blood and may be direct consequences of infection or biomarkers of tissue pathology in COVID-19 [10,11].

The early identification of patients with poor prognoses would help clinicians to manage the clinical therapeutic options. Here, we propose a model including easily measurable immunological parameters to predict the patients at risk of worse outcomes.

## 2. Materials and Methods

### 2.1. Patients and Blood Sampling

The Regional Ethics Committee (CEIm, internal code 2020.167, 14 May 2020) approved the protocol for the patients included in the study. Patients at admission or, if not possible, a legal representative gave oral informed consent, which was expressed in the medical records. The inclusion criteria included subjects over the age of 18 years who demonstrated COVID-19 with positive RT-PCR for SARS-CoV-2. Disease severity was assessed based on their clinical records. The cohort was divided based on oxygen therapy requirements during their follow-up into those with no requirement (mild) and those who required oxygen therapy or intensive care or were deceased (moderate–severe). Blood was collected in sodium heparin tubes for flow cytometry and functional studies or tubes without additives for serum parameters at admission into the hospital.

### 2.2. Flow Cytometry for Main Peripheral Blood Lymphocytes

Frequencies and absolute numbers of CD3^+^, CD4^+^, CD8^+^, CD19^+^, CD16^+^/56^+^, and CD3^+^/CD16^+^/56^+^ were estimated using AQUIOS CL. (Beckman Coulter, Brea, CA, USA) volumetric flow cytometer. The instrument employs a volumetric approach for enumerating specific cell populations without the need for reference beads. Fifty microliters of whole blood from EDTA tubes was stained with CD45-fluorescein isothiocyanate (FITC), CD4-RD1, CD16-CD56-RD1, CD8-ECD, CD19-ECD, and CD3-phycoerythrin-cyanine 5 (PC5) (Beckman Coulter). After lysis, the sample was acquired in the automated “load and go” flow cytometer.

### 2.3. Flow Cytometry for B and T Cell Subsets and Monocyte Subpopulations

Peripheral blood mononuclear cells (PBMCs) were obtained by Ficoll Histopaque 1077 (Sigma Aldrich, St. Louis, MI, USA) gradient centrifugation. Briefly, PBMCs were freshly stained and processed following standard procedures. The following monoclonal antibodies were used to identify the different T lymphocyte subsets: anti-CD8-FITC clone B9.11 (Beckman Coulter), CD127-FITC clone R34.34, CD28-FITC clone CD28.2, CXCR3-FITC clone G025H7 (BioLegend, San Diego, CA, USA), CD25-phycoerythrin (PE) clone B1.49.9 (Beckman Coulter, Brea, CA, USA), HLA-DR-PE clone Immu-357, CD62L-ECD clone DREG56, CD45RO-ECD clone UCHL1, CD4-phycoerythrin-cyanine 5.5 (PC5.5) clone 13B8.2, CD27-phycoerythrin-cyanine 7 (PC7) clone 1A4CD27, CD38-allophycocyanin (APC) clone LS198-4-3, CD45RA-Alexa fluor 700 (AF700) clone 2H4LDH11 LDB9, CD3-pacific blue (PB) clone UCHT1, and CD45-Krome orange (KrO) clone J33.

The different B-lymphocyte subsets were identified using the following monoclonal antibodies: anti-IgD-FITC clone IA6-2 (Beckman Coulter), CD27-PC5.5 clone 1A4CD27, CD19-PC7 clone J3-119, and CD45-KrO clone J33.

The following monoclonal antibodies were used to identify the different monocyte subpopulations: anti-CD14-PE clone RMO52 (Beckman Coulter), CD16-APC clone 3G8, and CD45-KrO clone J33.

### 2.4. TLR Protein Expression in PBMCs

The cell-surface expression of TLR4 and the intracellular expression of TLR3, TLR7, and TLR8 were assessed in different PBMC subpopulations including T lymphocytes, B lymphocytes, and monocytes by flow cytometry, as previously shown [12]. PBMCs collected into EDTA tubes were isolated by Ficoll Histopaque 1077 and stained with CD3-PB clone UCHT1 (Beckman Coulter), CD19-PC5.5 clone J3-119, and CD14 ECD clone RMO52 to identify T lymphocytes, B lymphocytes, and monocytes, respectively, and with PE-conjugated anti-human TLR4 (eBioscience, San Diego, CA, USA) or PE mouse IgG2a isotype control for 20 min in the dark. To determine the intracellular expression of TLR3 (MiltenyiBiotec, Bergisch Gladbach, Germany), TLR7 (Abcam, CA, USA), and TLR8, cells were permeabilized with FACS permeabilizing solution (BD Bioscience, San Jose, CA, USA) and stained with PE-conjugated anti-human TLR or mouse isotype control for 20 min in the dark. Expression of TLRs was assessed by flow cytometry (Navios, Beckman Coulter).

### 2.5. SARS-Cov2 T-Specific Response Assessment by Flow Cytometry

The procedure was validated by the Spanish Society of Immunology and based on activation-induced marker (AIM) expression after exposure to specific SARS-CoV-2 antigens [13]. Briefly, the PBMCs from heparinized blood were isolated by Ficoll gradient and cultured at 10^6^ cells/mL in TexMACS medium (MiltenyiBiotec) for 24 h at 37 °C in a flat-bottom 96-well plate in 0.1% DMSO; PepTivator SARS-CoV-2 Prot S, Prot M, and Prot N (1 µg/mL); and Dynabeads Human T activator CD3/CD28 (Gibco Thermo Fisher Scientific Baltics UAB, Vilnius, Lithuania) as a polyclonal stimulus. After incubation, the PBMCs were washed and stained with the following monoclonal antibodies: anti-CD3 (FITC) clone UCHT 1 (Inmunotech SAS Beckman Coulter, Marseille, France), anti-CD134 (PE) clone 134-1 (Cytognos, Salamanca, Spain), anti-CD8 (ECD) clone SFCI21Thy2D356,22,23 (Beckman Coulter), anti-CD25 (PE-CyTM7) clone 2A3, and anti-CD4 (APC-Vio 770) clone VIT4 (MiltenyiBiotec, Bergisch Gladbach, Germany). The stained PBMC samples were washed with PBS 150 µL and centrifuged for 5 min at 1800 rpm. Finally, 2 µL of 7-Aminoactinomycin D (7-AAD) staining solution (Tonbo Biosciences, San Diego, CA, USA) and 90 µL of PBS were added before the samples were acquired on the CytoFLEX Flow Cytometer (Beckman Coulter). Results were expressed as the ratio of the frequency in the AIM obtained after specific activation to negative non-stimulated control. A ratio >3 in one of the specific SARS-CoV-2 peptides was considered as a positive reaction.

### 2.6. Determination of Circulating IL-6

Human IL-6 was measured by ELISA (Enzo Life Sciences, Inc., Farmingdale, NY, USA) following the manufacturer’s instructions. The sensitivity of IL-6 serum levels was 0.057 pg/mL. Intra- and interassay variability were 4.38% and 9.6%, respectively.

### 2.7. Statistical Analysis

Statistical analysis was performed using Graph Pad Prism software. The distribution of continuous variables was assessed using Kolmogorov–Smirnov/Shapiro–Wilk tests where indicated. Results were expressed as mean ± standard deviation or median + interquartile range (IQR) for continuous variables and percentages for categorical data. Comparisons were based on the unpaired T-Student test or U-Mann–Whitney U test for parametric and nonparametric continuous data, respectively. Welch correction was applied when appropriate. A two-sided *p*-value < 0.05 was considered statistically significant. In order to identify variables associated with moderate–severe clinical outcomes, logistic regression analysis was performed. After univariate analysis with the potentially independent variables, the odds ratio was calculated with Wald’s statistic. In a further multivariate analysis, those with *p* < 0.25 value in the univariant analysis, following the proposed Hosmer and Lemeshow criteria [14], and supported by other reference authors [15] were included in the analysis. For the model selection, the backward method procedure was used to perform automatically variable selection. To assess the predictive capability of the model, the area under the curve (AUC) was used.

## 3. Results

### 3.1. Patient Demographics and Baseline Characteristics at COVID-19 Onset

One hundred and fifty-five COVID-19-positive patients recruited during the first days after hospital admission (mean of 1.0, interquartile range (IQR) (1–2) days of admission) were included in the study from April–October 2020. The median of days between the onset of symptoms and admission was 6 days (IQR 3–9).

The cohort was divided according to their clinical progression after admission into two groups: patients without oxygen therapy (73 included in the mild disease group) and those with oxygen therapy requirements (82 included in the severe disease group). The patients with severe disease were significantly older and had lower oxygen saturation at admission than the mild-disease group. The levels of C-reactive protein (CRP), troponin, ferritin, lactate-dehydrogenase (LDH), C4, and IL-6 were significantly higher in severe patients. The D-dimer levels were also increased in the severe group, although not significantly. No changes in serum concentration of immunoglobulins (IgG, IgA, and IgM) at admission were observed between mild and severe groups, and the concentration remained within the normal range values. Table 1 summarizes the main demographic, analytical, and clinical parameters compared between groups.

### 3.2. Innate-Immune Compartment Assessment at Admission

**Figure 1:**
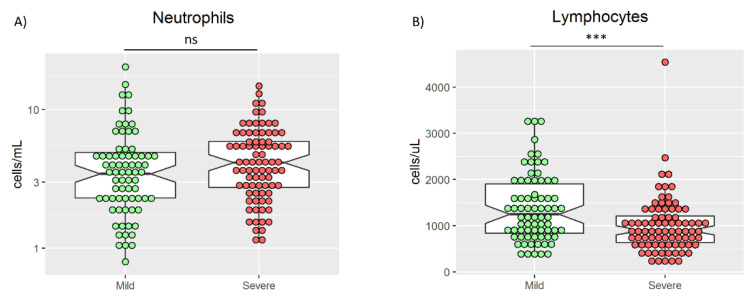
Absolute count of neutrophils (**A**) and lymphocytes (**B**) in mild and severe patients. U Mann–Whitney test was used to compare medians in A and B. ***: *p* < 0.001. ns: not significant.

**Figure 2:**
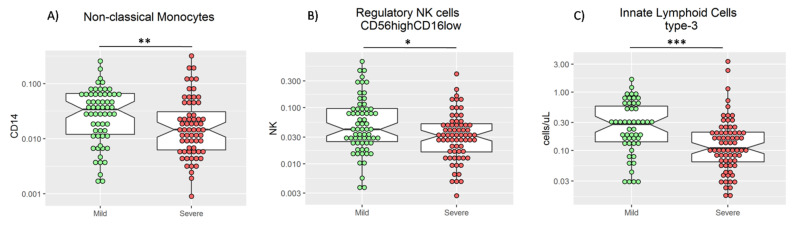
Frequency of non-classical monocytes (**A**) and regulatory NK cells (**B**), and absolute count of innate lymphoid cells type-3 (**C**) in mild and severe patients. U-Mann–Whitney test was used to compare medians in (**A**–**C**), * *p* < 0.05, ** *p* < 0.01, and *** *p* < 0.001.

The innate immune system is involved in the first stage of any viral infection, including COVID-19 disease [16]. The main cellular components of the innate immunity to be measured in peripheral blood are neutrophils, monocytes, NK, and innate lymphoid cells (ILC). In patients with active COVID-19 disease, different innate immune signatures have been identified from mild to severe disease [16,17]. Those patients with a more severe phenotype had increased neutrophil and reduced monocyte frequency at admission [18,19]. In our cohort, these data are confirmed (Table 1, Figure 1A). Moreover, a significant increase in the percentage of non-classical monocytes in the mild group was observed (*p* = 0.01; Figure 2A). In addition, within the innate lymphoid cells (ILC), a significant increase in both the frequency of regulatory NK (CD3^−^CD56^high^CD16^−/low^) cells (*p* = 0.016, Figure 2B) and the absolute number of ILC type-3 (*p* < 0.001) in the mild group was observed (Figure 2C).

Toll-like receptors (TLRs) are important innate immune receptors in recognizing viral particles and play an essential role in the induction of the first line of immune responses. Among the TLRs described in humans, TLR3 and TLR7 have been involved in the immune response against SARS-CoV-2 [20,21]. Therefore, the expression of TLR3, TLR7, and TLR4, as control, was measured. However, no differences in TLR expression between the two groups of patients were found (Table 2).

### 3.3. Adaptive Immune Compartment Assessment at Admission

**Figure 3:**
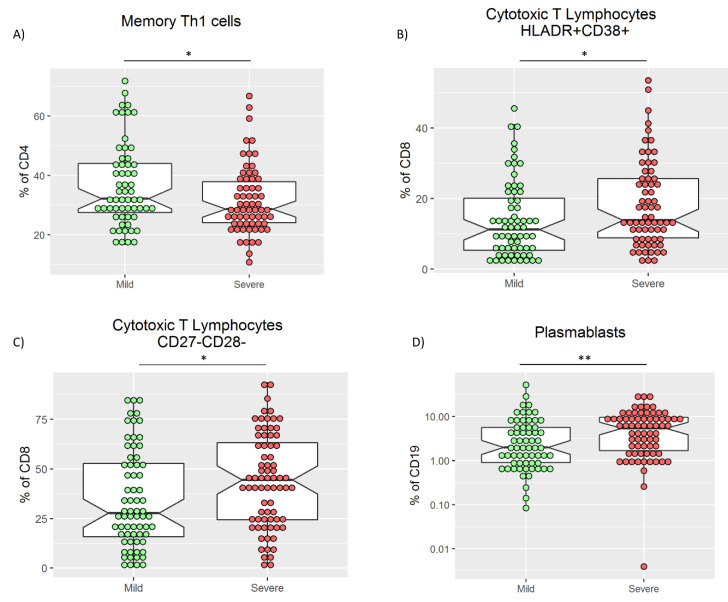
Frequency of memory Th1 cells (**A**), cytotoxic T lymphocytes HLADR^+^CD38^+^ (**B**), cytotoxic T lymphocytes CD27^−^CD28^−^ (**C**), and plasmablasts (**D**) in mild and severe patients. U-Mann–Whitney test was used to compare medians in A, B, C, and D. * *p* < 0.05 and ** *p* < 0.01.

As previously described [22,23], marked lymphopenia in severe patients was confirmed (Table 1). To avoid skew interpretation in absolute counts, only relative frequencies were evaluated. In the main lymphocyte subsets, a significantly higher percentage of T lymphocytes at admission with a reduction of B and NK cells in mild patients was observed compared with the severe group. No differences were observed in the frequency of CD4 and CD8 T cell subsets (Table S1). Within the CD4 T cell compartment, an increase in the frequencies of both total Th1 (CD4^+^CXCR3^+^CCR6^−^) and memory Th1 (CD4^+^CD45RO^+^CXCR3^+^CCR6^−^) T cells in the mild group was observed (*p* = 0.057 and *p* = 0.030, respectively, Table 3, Figure 3A). Notably, the frequency of peripheral blood T cells with a regulatory phenotype (Tregs) in mild patients was slightly higher than in severe patients (*p* = 0.063) (Table 3).

Conversely, the CD8^+^ T cells are cytotoxic antiviral lymphocytes, and an increased proportion of activated and exhausted CD8^+^ T cells has been described in COVID-19 [24]. Accordingly, we found a significantly increased frequency of CD8^+^CD38^+^HLA-DR^+^ (Figure 3B) and CD8^+^CD27^−^CD28^−^ in the severe group compared with the mild group (Table 3, Figure 3C). On the other hand, the frequency of naïve CD8 populations CD8^+^CD62L^+^CD45RA^+^ and CD8^+^CD27^+^CD28^+^ increased in the mild group. Finally, the frequency of effector population CD8^+^CXCR3^+^CCR6^+^ and memory CD8^+^CD45RO^+^CXCR3^−^CCR6^+^ were increased in the mild group at admission (Table 3).

In addition, as previously described [19], a significantly high proportion of plasmablasts (CD19^+^CD20^−^CD27^high^CD38^high^) in the severe group was confirmed (Table 3, Figure 3D).

### 3.4. SARS-CoV-2 Specific T Cells Response in Active COVID-19 Disease

Phenotypic characterization of immune cells may not reflect their function and specificity. The specific T cell response against overlapping peptide pools of the nucleocapsid phosphoprotein (“N”), the membrane glycoprotein (“M”), and the surface glycoprotein (“S”) of SARS-CoV-2 through activation of PBMC in both mild and severe groups was assessed. The response was evaluated by expressing activation-induced markers (CD134 and CD25) after 24 h of stimulation, as previously shown [13]. Anti-CD3/CD28 monoclonal antibody stimulation was used as a positive control, while medium without additives was used as a negative control. The global stimulation index with any SARS-CoV-2 antigen was comparable between groups at admission (Table S2).

### 3.5. Assessment of the Immune Parameters as a Prognosis Factor

**Figure 4:**
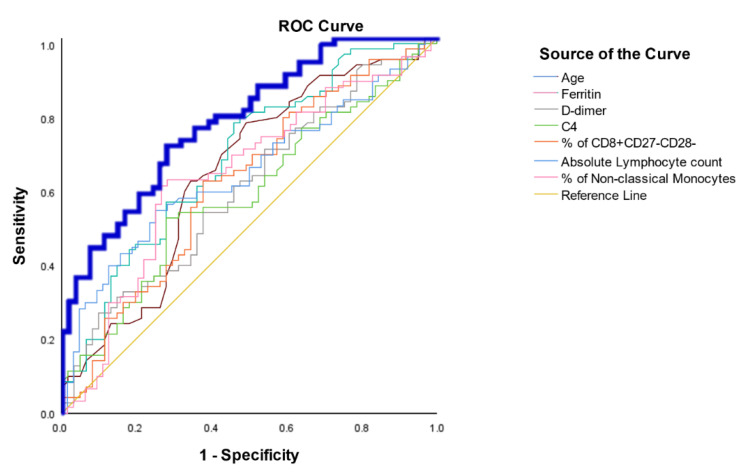
ROC curve analysis of the logistic regression model for prediction of moderate–severe outcomes. Superposed and independent AUC analyses of the variables included in the logistic regression model are depicted. The AUC of the merged probability is calculated from the predictive model in bold blue line as shown in the Materials and Methods section.

Within all the evaluated immune parameters included in this study, those with significant differences at admission were selected in order to investigate their independent role in the prognosis of the patients. The univariate and multivariate analyses are summarized in Table 4. The logistic regression model was performed as described in Materials and Methods, and the parameters finally included in the model were: age, ferritin, D-dimer, absolute counts of lymphocytes, C4, CD8^+^CD27^−^CD28^−^, and non-classical monocytes. The area under the curve was 78.2%, with a sensitivity and specificity of 71.4 and 72.2, respectively (Figure 4).

## 4. Discussion

The COVID-19 disease has been divided into two well-differentiated stages, firstly an inflammatory step and subsequently a hyper-inflammatory step. The inflammatory response is conducted by innate immune components early after SARS-CoV-2 infection. An average of 10 days has been estimated for this response, followed by the induction of an efficient adaptive specific response in mild disease. However, if this immune response is overcome, a further hyper-inflammatory response is mounted. This hyper-inflammatory response has been associated with severe and poor clinical outcomes [25,26,27].

Different immune profiles at admission have been associated with clinical outcomes, underlining the presence of lymphopenia [10], neutrophilia [11], and an increase in monocyte subsets [28]. Furthermore, alterations in adaptive immune system components, including activated and exhausted phenotypes in cytotoxic T cells, have been confirmed [29]. Moreover, increased levels of plasmablasts in severe patients have been observed [19].

A comprehensive immune profile was created in the present work, and the obtained results were comparable with those described in previous studies [28,29] (Table 2 and Table 3). Among differential features in the innate immune system in severe versus mild COVID-19 patients, a dysfunctional neutrophil skew was observed in severe cases [30]. This emergency myelopoiesis could be associated with an increased frequency of neutrophils and lymphopenia, as observed in our cohort. Moreover, non-classical monocytes were expanded within the monocyte compartment in the mild group compared with severe cases [31]. This monocyte subset has been involved in inflammation restoration and tissue recovery [32], whereas its increased frequency in mild patients could be related to virus clearance [33].

The role of total ILCs and specifically ILC1 in antiviral immune response has been previously shown [34,35]. A reduction of ILC1 in severe COVID-19 patients has been recently described by García et al. [35]. Our results confirmed the reduction of this cell subset in severe COVID-19 patients, although no significant differences were observed.

In contrast, the role of ILC3 in respiratory viral infections has not been described yet. ILC3 exist mainly in the intestinal mucosal tissue, playing an important function in mucosal homeostasis and inflammatory responses. Nevertheless, we observed a significant reduction of this subpopulation in severe COVID-19 patients at admission. The function of ILC3 in the intestinal mucosa is well described [36]. Their role in respiratory mucosa remains to be elucidated. Nonetheless, our finding was in peripheral blood, and the relationship between circulating and tissue ILC3 is not established yet.

TLR signaling in viral infections has been thoroughly studied. Specifically, TLR-3, TLR-7, and TLR-8 exert a key role in infections by RNA viruses, such as SARS-CoV-2. Functional studies have identified rare loss-of-function variants of the X-chromosomal *TLR7* in severe COVID-19 patients [37]. In our cohort, the patients with severe disease were older and had several comorbidities that could go unnoticed, such as a loss-of-function effect, since no significant differences in the expression of TLR-3 and TLR-7 considering the severity of COVID-19 patients were observed.

The potential role of regulatory subsets in COVID-19 prognosis was studied by Meckiff et al. [38]. They observed a skew towards a reactive gene expression pattern of SARS-CoV-2-specific CD4^+^ T cells with impairment of Tregs in severe patients. In our cohort at admission, the severe patients had reduced Treg frequency and CD3^−^CD56^++^CD16^lo^ NK cells compared with the mild group [39].

The Th1 response is involved in cellular immunity throughout IFN-γ production. In our cohort, an increase in Th1 and memory Th1 cells was observed in the mild group. This observation points to an early activation compared with the severe group. Previous studies on Th subsets in COVID-19 have shown poor outcomes related to undifferentiated Th subsets in patients [40] or with a skew towards Th2 cells [41].

The cytotoxic T cell is the main subset in adaptive antiviral response. After immunophenotype analysis, the CD8 compartment has been classified in detail not only by maturation stage but also activation status. The early activation phenotype in CD8 is defined by CD38 and HLA-DR expression [42]. Recently, an increase in the frequency of CD8^+^CD38^+^HLADR^+^ cells in patients with COVID-19 disease and fatal outcomes has been confirmed [43,44]. In the present work, the severe group presented an increased frequency of CD8^+^CD38^+^HLADR^+^ cells at admission. In terms of the functional status of CD8 cells, severe patients had an exhausted or immunosenescence phenotype [45]. We used CD27 and CD28 to identify a CD8 exhausted phenotype [29], and accordingly, a significant increase in the CD8^+^CD27^−^CD28^−^ exhausted phenotype in the severe group was found.

This exhausted phenotype of CD8^+^ T cells was included in the predictive model to establish the risk of severe disease. Together with age, IL-6, ferritin, D-dimer, IgM, C4, absolute lymphocyte count, ILC type-3 count, and percentage of plasmablasts, Th1, memory Th1, Treg, CD8^+^CD38^+^HLA-DR^+^, non-classical monocytes, and CD3^−^CD56^++^CD16^lo^ NK cells, the predictive model showed an AUC of 78%. Several predictive models have been published based on demographic, biochemical, and immunological parameters [39,46,47,48,49]. The main prognostic factors were neutrophil and lymphocyte counts, whereas NK subsets and CD4 levels were only partially confirmed. Notably, other associated parameters with poor prognosis in COVID-19 patients such as SARS-CoV-2 viral load have been demonstrated [50]. A limitation of the study was the absence of the viral load or the cycle threshold (Ct) data in our model.

Easily measurable immune parameters, such as CD8^+^CD27^−^CD28^−^ and non-classical monocytes, improve the predictive value of our model. However, the cross-sectional design is a limitation of the study, and further validation cohorts should be assessed to confirm the model’s predictive capability.

Although our model was not developed to predict fatal outcomes, both innate and adaptive immune parameters could help determine the oxygen therapy requirement of 78% of the patients and could be helpful in improving the therapeutic management of the patients at admission.

## Figures and Tables

**Table 1:** Demographic, analytical, and clinical parameters.

	Mild (*n* = 73)	Moderate–Severe (*n* = 82)	*p*-Value	Reference Values
Demographic				
Age (years)	59 (47–77)	72 (63–79)	<0.001	NA
Gender (% female)	43 (58.90%)	26 (31.71%)	0.001	NA
Comorbidities				
Hypertension	30 (41.10%)	43 (52.44%)	NS (0.158)	NA
Type II diabetes	11 (15.07%)	17 (20.73%)	NS (0.360)	NA
Heart disease	12 (16.44)	20 (24.39%)	NS (0.222)	NA
Respiratory disease	6 (8.22%)	8 (9.76%)	NS (0.739)	NA
Obesity	12 (16.44)	11 (13.41%)	NS (0.597)	NA
Biochemical parameters				
C-reactive protein (mg/dL)	2.9 (0.9–6.6)	6.5 (3.0–10.7)	0.001	0.1–0.5
Ferritin (ng/mL)	203.5 (105.5–603)	535 (224–1135)	<0.001	10–291
D-dimer (ng/mL)	540 (313–992)	702 (389–1309)	NS (0.199)	0–500
Troponin (ng/mL)	5 (3–14)	11 (6–21)	0.006	0–40
LDH (IU/L)	227 (173–277)	274 (223–362)	<0.001	120–246
O_2_ saturation at admission (%)	97 (96–98)	95 (93–97)	<0.001	NA
Complete blood count				
Lymphocytes (%)	23.40 (16.00–32.75)	16.65 (10.80–24.90)	0.001	20.0–50.0
Neutrophils (%)	64.85 (54.35–74.40)	74.00 (65.30–81.40)	<0.001	42.0–75.0
Monocytes (%)	8.70 (6.85–11.85)	7.05 (4.70–10.00)	0.003	2.0–13.0
Lymphocytes count (×10^3^)	1.20 (0.80–1.80)	0.90 (0.70–1.20)	0.001	1.2–5.0
Neutrophils (×10^3^)	3.45 (2.30–4.90)	4.15 (2.70–5.90)	NS (0.077)	1.4–7.5
Monocytes (×10^3^)	0.53 ± 0.27	0.45 ± 0.26	0.051	0.2–1.0
Serum immune factors				
IgG (mg/dL)	1094.91 ± 351.20	1096.39 ± 344.30	NS (0.979)	734–1486
IgM (mg/dL)	98.18 (73.85–134.31)	82.68 (51.42–133.88)	NS (0.078)	41–201
IgA (mg/dL)	262.36 ± 155.21	279.47 ± 135.86	NS (0.454)	49–401
C3 (mg/dL)	131.50 ± 33.02	133.32 ± 30.60	NS (0.724)	77–203
C4 (mg/dL)	31.04 (25.26–37.02)	35.44 (27.86–40.22)	0.019	7.7–50.5
IL-6 (ng/dL)	26.68 (8.12–54.20)	33.88 (7.46–125.0)	0.048	0–30

Abbreviations: LDH: lactate dehydrogenase; NA: not applicable; NS: not significant. For parametric and nonparametric variables, mean ± SD and median (interquartile range) are shown. For comparison, T-Student and U-Mann–Whitney test, respectively, were used. The comparison of frequencies was addressed by the Chi-square test.

**Table 2:** Comparison of innate immunity parameters in mild and moderate–severe COVID-19 patients.

	Mild (*n* = 73)	Moderate–Severe (*n* = 82)	*p*-Value
Monocytes			
Classic (%CD14^+^CD16^−^)	70.34 (55.9–79.6)	71.1 (49.5–82.2)	NS (0.896)
Intermediate (%CD14^+^CD16^+^)	27.9 (17.4–39.9)	27.0 (15.2–42.9)	NS (0.677)
Non-classic (%CD14^−^CD16^+^)	3.4 (1.2–6.6)	1.5 (0.6–3.5)	0.010
TLR expression			
TLR3	1.1 (0.8–1.7)	1.1 (0.9–1.6)	NS (0.956)
TLR4	2.1 (1.0–3.1)	1.7 (1.1–2.6)	NS (0.593)
TLR7	1.4 (1.0–2.3)	1.3 (1.0–2.1)	NS (0.631)
NK cells			
%CD16/56	13.77 (10.71–23.1)	17.25 (11.7–25.9)	NS (0.097)
%NKT	5.05 (3.205–10.945)	4.52 (3.76–9.25)	NS (0.746)
^#^ CD16/56	169 (114–277)	159 (98–230)	NS (0.205)
^#^ NKT	59 (32.5–130)	44 (30–71)	0.019
CD56^+^CD16^−^	4.2 (2.5–9.7)	3.2 (1.6–5.1)	0.014
CD56^+^CD16^+^	95.8 (90.3–97.5)	96.8 (94.8–98.3)	0.014
ILCs			
ILC1 (Lin^−^CD127^+^CD117^−^CD294^−^)	2.33 (1.33–4.64)	1.66 (0.94–3.88)	NS (0.198)
ILC2 (Lin^−^CD127^+^CD117^+^CD294^+^)	0.32 (0.13–0.73)	0.25 (0.11–0.44)	NS (0.497)
ILC3 (Lin^−^CD127^+^CD117^+^CD294^−^)	0.28 (0.14-0.60)	0.11 (0.06-0.21)	0.00028

Abbreviations: TLR: Toll-like receptor; NK: natural killer; NKT: natural killer T cells; ^#^: absolute count (cells/µL); ILC: innate lymphoid cells; NS: not significant. For parametric and non-parametric variables, mean ± SD and median (interquartile range) are shown. For comparison, T-Student and U-Mann–Whitney tests, respectively, were used. All TLR expression was calculated as the ratio of MFI of specific TLR monoclonal Ab/isotype control. See Materials and Methods for details.

**Table 3:** Comparison of frequencies of T and B lymphocyte functional subsets between groups.

	Mild (*n* = 73)	Moderate–Severe (*n* = 82)	*p*-Value
T helper subsets (CD4^+^)			
CD4^+^CD27^+^CD28^+^	86.7 (73.9–93.9)	87.1 (75.2–93.6)	NS (0.782)
CD4^+^CD27^−^CD28^+^	4.8 (3.1–8.1)	4.3 (2.5–7.2)	NS (0.695)
CD4^+^CD27^+^CD28^−^	0.6 (0.3–1.0)	0.8 (0.3–1.2)	NS (0.724)
CD4^+^CD27^−^CD28^−^	6.5 (1.0–16.8)	6.7 (2.0–13.6)	NS (0.927)
CD4^+^CXCR3^+^CCR6^−^ (Th1)	23.9 (18.3–34.8)	20.1 (15.3–30.0)	0.057
CD4^+^CXCR3^+^ (Th1/Th17)	12.6 (8.7–16.0)	9.6 (7.1–14.0)	0.039
CD4+CXCR3^−^CCR6^+^ (Th17)	12.3 ± 5.0	12.4 ± 5.2	NS (0.907)
CD4^+^CD45RO^+^ (Memory Th)	62.8 (50.4–71.9)	58.1 (40.2–72.0)	NS (0.064)
CD4^+^CD45RO^−^CD62L^+^ (Naïve)	19.7 (12.7–29.3)	18.5 (9.9–31.3)	NS (0.290)
CD4^+^CD45RO^+^CD62L^+^ (TCM)	46.4 ± 13.9	48.3 ± 15.8	NS (0.234)
CD4^+^CD45RO^+^CD62L^−^ (TEM)	24.5 (17.8–38.5)	21.3(11.5–42.8)	NS (0.252)
CD4^+^CD45RO^−^CD62L^−^ (TEMRA)	1.4 (0.5–3.8)	1.3 (0.6–3.9)	NS (0.957)
CD4^+^CD45RO^+^CXCR3^+^CCR6^−^(Memory Th1)	32.2 (26.9–44.4)	28.7 (24.0–38.0)	NS (0.030)
CD4^+^CD45RO^+^CXCR3^+^ (Memory Th1/Th17)	19.4 (16.1–25.2)	23.2 (17.5–26.0)	NS (0.137)
CD4^+^CD45RO^+^CXCR3^−^CCR6^+^ (Memory Th17)	21.1 ± 8.7	18.5 ± 7.9	NS (0.098)
CD4^+^CXCR3^−^CCR6^−^CD294^+^ (Th2)	1.0 (0.7–1.7)	0.8 (0.4–1.3)	NS (0.830)
CD4^+^CD45RO^+^CXCR5^+^PD1^+^ (Tfh)	0.2 (0.1–0.4)	0.3 (0.1–0.5)	NS (0.153)
CD4^+^CD127^−^CD25^+^ (Tregs)	6.4 (5.5–7.5)	5.7 (4.3–7.2)	NS (0.063)
T cytotoxic subsets (CD8^+^)			
CD8^+^CD27^+^CD28^+^	57.1 (31.4–71.1)	37.6 (21.5–53.2)	0.004
CD8^+^CD27^−^CD28^+^	2.1 (1.2–3.7)	2.2 (1.1–3.7)	NS (0.580)
CD8^+^CD27^+^CD28^−^	10.2 (7.4–16.2)	12.0 (6.5–19.0)	NS (0.219)
CD8^+^CD27^−^CD28^−^	27.7 (15.8–53.1)	44.5 (24.4–63.2)	0.019
CD8^+^CXCR3^+^ (Tc1/Tc17)	4.9 (3.2–9.5)	3.0 (1.8–4.6)	0.0003
CD8^+^CD45RO^+^ (Memory Tc)	42.9 (34.9–57.7)	42.2 (35.2–57.6)	NS (0.749)
CD8^+^CD45RO^−^CD62L^+^ (Naïve)	25.9 (14.8–40.8)	19.2 (10.3–28.8)	0.026
CD8^+^CD45RO^+^CD62L^+^ (TCM)	15.0 (10.0–19.2)	14.1 (8.8–21.7)	NS (0.942)
CD8^+^CD45RO^+^CD62L^−^ (TEM)	30.9 (23.9–38.7)	31.6 (22.6–44.6)	NS (0.780)
CD8^+^CD45RO^−^CD62L^−^ (TEMRA)	21.0 (11.8–34.4)	26.1 (14.3–38.1)	NS (0.125)
CD8^+^CD45RO^+^CXCR3^+^ (Memory Tc1/Tc17)	2.5 (1.4–6.6)	2.8 (1.2–5.1)	0.0002
CD8^+^DR^+^CD38^+^	11.2 (5.3–20.5)	13.8 (8.8–25.6)	0.028
B lymphocytes			
B naïve (CD27^−^IgD^+^)	65.3 (47.8–75.5)	63.8 (48.3–75.0)	NS (0.656)
B unswitched (CD27^+^IgD^+^)	15.4 (9.0–23.4)	11.5 (8.3–21.5)	NS (0.196)
B switched (CD27^+^IgD^−^)	15.9 (8.5–24.1)	17.0 (9.8–25.5)	NS (0.478)
Plasmablasts (CD19^+^ CD20^low^CD27^hi^ CD38^hi^)	1.9 (0.8–5.8)	5.3 (1.6–9.7)	0.002

Abbreviations: Th: helper T cell; TCM: central memory T cells; TEM: effector memory T cells; TEMRA: terminally differentiated T cells; Tregs: regulatory T cells; Tc: cytotoxic T cells; Tfh: T follicular helper cells; NS: not significant. For parametric and non-parametric variables, mean ± SD and median (interquartile range) are shown. For comparison, T-Student and U-Mann–Whitney tests, respectively, were used.

**Table 4:** Univariate and Multivariate analysis of the parameters included in the logistic regression model.

Parameter		Univariate			Multivariate	
	*p*	Odds	CI	*p*	Odds	CI
Age	<0.001	1.033	1.013–1.053	0.015	1.038	1.007–1.069
Ferritin	<0.001	1.001	1.001–1.002	0.021	1.001	1.001–1.002
D-dimer	0.226	1.000	1.000–1.000	0.01	1.000	1.000–1.001
Absolute lymphocyte count	0.002	0.999	0.999–1.000	0.023	0.999	0.998–1.000
C4	0.016	1.041	1.007–1.075	0.110	1.036	0.992–1.082
% of CD8^+^CD27^−^CD28^−^	0.023	1.017	1.002–1.031	0.701	1.004	0.985–1.023
% of non-classical monocytes	0.288	0.18	0.000–29.826	0.908	1.712	0.000–0.000149

Abbreviations: CI: confidence interval.

## Data Availability

All data generated or analyzed during this study are included in the published article.

## Supplementary Materials

The following are available online at https://www.mdpi.com/article/10.3390/biomedicines9080917/s1: Table S1: Comparison of main lymphocyte subsets expressed as frequencies and absolute numbers in peripheral blood. Table S2: CD134 and CD25 expression after stimulation with N-, M-, and S-specific SARS-CoV-2 antigens.
