# Supplementary material for: Innate and Adaptive Immune Assessment at Admission to Predict Clinical Outcome in COVID-19 Patients
Source: Biomedicines. 2021 Jul 29;9(8):917. doi: 10.3390/biomedicines9080917 (PMC8389676; doi:10.3390/biomedicines9080917)
Supplement: Supplementary file 1 [file biomedicines-09-00917-s001.zip › biomedicines-1292086-supplementary.pdf]

Table S1. Comparison of main lymphocyte subsets expressed as frequencies and absolute numbers in peripheral blood. Values are shown as Median (IQR) and Mean ± SD.

	**Mild (n=73)**	**Moderate-Severe (n=82)**	**p-value**
**%CD3**	70.30±10.79	65.81±13.11	0.022
**%CD4**	45.33±13.2	42.02±12.93	NS (0.118)
**%CD8**	21.76±11.74	20.18 ±11.60	NS (0.403)
**Ratio**	2.15 (1.39-3.74)	2.32 (1.36-3.83)	NS (0.498)
**%CD19**	10.56±6.29	11.94±10.30	NS (0.324)
**#Lymphocytes**	1249 (836-1909)	904 (639-1216)	0.001
**#CD3**	866 (534-1406)	619 (367-839)	< 0.001
**#CD4**	553 (383-857)	358 (228-544)	< 0.001
**#CD8**	227 (122-476)	156 (86-297)	0.007
**#CD19**	105 (62-200)	82 (45-122)	0.762

For parametric and non parametric variables, mean ± SD and median (interquartile range) are shown. For comparison, T-student and U-Mann Whitney test, respectively were used.
Abbreviations: IQR: Interquartile Range; SD: Standard Deviation; NS: Not Significant. # Absolute count.

Table S2. CD134 and CD25 expression after stimulation with N, M and S specific SARS-CoV-2 antigens

	Mild (n=38)	Moderate-Severe (n=42)	p-value
CD4; median (IQR)			
**N**	1.43 (0.82-2.96)	1.67 (1.04-2.80)	NS (0.305)
**S**	1.61 (0.83-3.20)	1.94 (1.62-3.01)	NS (0.551)
**M**	1.64 (0.99-3.11)	1.77 (1.03-2.90)	NS (0.380)

U-Mann Whitney test for comparison was applied.
Abbreviations: HC, healthy controls; WS, without stimulation; N, nucleocapsid peptide; S, spike peptide; M, membrane peptide; Ag, antigen.
